# Association of major dietary patterns with obesity, hypertension, and cognitive function in older adults: A cross-sectional study

**DOI:** 10.34172/hpp.025.43640

**Published:** 2025-07-15

**Authors:** Arezou Akhbari, Sevil Kiani, Sina Naghshi, Mahtab Rajabi-Jourshari, Hamid Allahverdipour, Maryam Saghafi-Asl

**Affiliations:** ^1^Research Center for Integrative Medicine in Aging, Tabriz University of Medical Sciences, Tabriz, Iran; ^2^Student Research Committee, Tabriz University of Medical Sciences, Tabriz, Iran; ^3^Department of Health Education and Promotion, Faculty of Health, Tabriz University of Medical Sciences, Tabriz, Iran; ^4^Department of Clinical Nutrition, School of Nutrition and Food Sciences, Tabriz University of Medical Sciences, Tabriz, Iran

**Keywords:** Aged cognition, Dietary patterns, Hypertension, Obesity abdominal, Obesity

## Abstract

**Background:**

There is limited data on the association between dietary patterns and health outcomes in older adults. The aim of this study was to investigate the association of major dietary patterns with obesity, hypertension, and cognitive function in this population. The aim of this study was to investigate the association of major dietary patterns with obesity, hypertension, and cognitive function in older adults.

**Methods:**

This cross-sectional study was performed on 337 participants aged 60 years or older. Dietary data were collected using a validated semi-quantitative food frequency questionnaire. Data regarding height, weight, waist circumference (WC), and blood pressure were collected using standard methods. Obesity was defined as body mass index (BMI)≥30 kg/m^2^, and abdominal obesity was defined as WC≥95 cm for men and women. Hypertension was defined as blood pressure≥140/90 mm Hg or taking anti-hypertensive medications. Mini-Mental State Examination (MMSE) score validated for Iranians, was applied to assess cognitive function. Dietary patterns were identified using factor analysis procedure.

**Results:**

Three major dietary patterns including mixed, healthy, and unhealthy were identified. There was an inverse association between the mixed dietary pattern and both abdominal obesity (odds ratio [OR]: 0.39, 95% confidence interval [CI]: 0.20-0.76) and general obesity (OR: 0.49, 95% CI: 0.24-0.99). A higher score of mixed pattern was also associated with lower odds of hypertension (OR: 0.39, 95% CI: 0.20-0.78). Moreover, a significant positive association was observed between unhealthy dietary pattern and hypertension (OR: 1.86, 95% CI: 1.01-3.43). A significant positive association was also observed between the unhealthy dietary pattern and abdominal obesity (OR: 1.90, 95% CI: 1.05-3.44).

**Conclusion:**

Our findings underscore that higher consumption of certain types of healthy foods (loaded strongly in mixed dietary pattern) could be a viable strategy for prevention of obesity and hypertension.

## Introduction

 The prevalence of obesity and hypertension has drastically increased worldwide.^1,2^ On the other hand, cognitive disorders have become global health problems along with other chronic conditions such as diabetes, cardiovascular disease, and cancer.^3^ Dietary modifications have long been documented as one of the most important approaches to prevent such diseases.^4-6^

 Several epidemiological investigations have assessed the association of diet with obesity, hypertension, and cognitive function in terms of individual nutrients,^7,8^ foods,^9,10^ and food groups.^11^ However, nutrients and foods are not consumed individually, but in combination with each other. Therefore, approaches that account for inter-relations of food intakes and represent the cumulative exposure to dietary components could reflect real-world diet-disease associations.^12^ Food patterns with such characteristics provide valuable information in guiding dietary modifications to reduce disease risk.

 Several studies have examined the relationship between dietary intakes and health outcomes, but these studies have mainly been conducted in young and middle-aged adults^13-17^ and limited data exist on elderly population, especially regarding dietary patterns. Neri-Sánchez et al^18^ conducted a study on healthy Mexican adults, which found that healthy dietary pattern was linked to a lower risk of central obesity. However, there was no significant association between risky and empty dietary patterns with central and general obesity. In a cross-sectional study involving 245 female university students, Western and high-protein dietary patterns were associated with higher and lower odds of general obesity, respectively.^19^ Moreover, participants in the last category of the healthy dietary pattern were less likely to be centrally obese. Among the three major dietary patterns (plant-based, high-protein, and unhealthy) identified in a prospective case-cohort study involving 294 participants with newly diagnosed hypertension and a representative random sub-cohort of 1,295 individuals, plant-based dietary pattern was inversely associated with a lower risk of hypertension.^20^ In the cohort of older adults in New Zealand, there was no significant association between Mediterranean style, Western, and Prudent dietary patterns with cognitive function.^21^ Taken together, studies have reported conflicting results.

 Due to physiological and health conditions, food intakes and dietary patterns are likely to be different in the elderly population, compared to other age groups. In addition, dietary intakes are specific and depend on the study population. For example, the dietary intakes and lifestyle factors of the Middle-East population are different from other parts of the world.^14^ In this region, high-energy-dense foods, hydrogenated vegetable oils, and refined carbohydrates such as rice and bread are consumed.^14^ Moreover, nutrition transition in the Middle-East countries is associated with a shift from traditional foods to Western diets. Furthermore, there is a unique pattern of obesity in this region, such that abdominal obesity is seen among men and women.^22^ Concerning the lack of studies that simultaneously investigate the relationship between major dietary patterns and multiple health outcomes—obesity, hypertension, and cognitive function—in a single, older adult population, it seems reasonable to investigate the association between diet and diseases in older people. Therefore, the current study was designed to examine the association of major dietary patterns with obesity, hypertension, and cognitive function in older adults. By investigating these associations collectively, our study provides additional perspective on the association of dietary patterns and aging-related health outcomes within Middle-East population.

## Materials and Methods

###  Participants

 This cross-sectional study was conducted in 2022, concerning older adults who were referred to urban and rural health centers in Jolfa, Iran. Participants were recruited using two-stage cluster sampling from different health centers in Jolfa. Health centers were selected randomly from different areas of Jolfa. A simple random sampling method was then applied within each center to randomly select participants, minimizing potential selection bias and enhancing the generalizability of the findings. Using a well-known formula for measuring sample size in cross-sectional studies^23^ and considering type one error (α) = 0.05, 68% prevalence rate,^24^ and precision (d) of 6%, 232 subjects were minimally required for this study.

 Considering the effect design of 1.4 and exclusion of participants with under- and over-reporting of dietary intakes, the final sample size of 345 participants was estimated. Eligibility criteria for the present analysis included age of 60 years or older,^25-27^ Iranian nationality, and having no special diet. Exclusion criteria included appetite change in the last month, hospitalization or any acute illness within the past three months, and suffering from neurological diseases. Participants with a history of cancer were also excluded because of possible disease-related changes in diet.

 After excluding participants who reported total energy intakes outside the normal range of 800–6000 kcal/d (n = 8),^28,29^ data from 337 older adults were included in the current analysis. The study protocol received approval from the Ethics Committee of Tabriz University of Medical Sciences in Tabriz, Iran (IR.TBZMED.REC.1401.575), and written informed consent was obtained from each participant.

###  Assessment of dietary intake

 Dietary intakes were assessed using an 80-item semi-quantitative food frequency questionnaire (FFQ) that was developed and validated for use in Iranian adults.^30^ A trained interviewer conducted face-to-face interviews to administer the FFQ. The daily intake of each food item was converted to grams per day based on household measures. Subsequently, the gram values of all food items were inputted into Nutritionist IV software (First Databank, San Bruno, CA, USA) to calculate the daily intake of energy and nutrients.

###  Assessment of anthropometric measures 

 Body weight was measured with an accuracy of 100 grams while fasting, without shoes, and wearing light clothes. Using a stadiometer (Seca, Hamburg, Germany), the height of the participants was measured without shoes, in a standing position, with an accuracy of 0.5 cm. Waist circumference (WC) was measured as the smallest horizontal circumference between the costal and iliac crests, using a non-stretchable measuring tape with 0.1 cm accuracy. Hip circumference was measured at the widest point above the great trochanters. Body mass index (BMI) was calculated by dividing weight in kilograms by the square of height in meters. General obesity was defined as BMI values equal to or greater than 30 kg/m^2^. Abdominal obesity was defined as WC values equal to or greater than 95 cm, which is a specified cutoff for the Iranian population.^31,32^

###  Assessment of cognitive function

 The cognitive function of the participants was evaluated using the validated Persian version of the Mini-Mental State Examination (MMSE) score.^33^ The MMSE evaluates seven cognitive function domains, which include time orientation, place orientation, registration, attention and calculation, recall, language, and visual construction. The total score ranges from 0 to 30 points, with higher scores indicating better cognitive function. For illiterate elderly participants, an alternative validated version of the MMSE was utilized.^34^

###  Assessment of blood pressure 

 Systolic and diastolic blood pressures (SBP and DBP) were measured twice with a 10-minute interval in a sitting position at the right arm using a mercury barometer, calibrated by the Institute of Standardization and Industrial Research. Before measurement, the participants were asked to rest for 5 minutes. The average of the two measurements was calculated and considered as participants’ SBP and DBP.^35^ Hypertension was defined as blood pressure ≥ 140/90 mmHg or taking anti-hypertensive medications.^36^

###  Assessment of other variables

 Data on lifestyle factors and medical history were gathered through in-person interviews using a comprehensive questionnaire. The questionnaire covered various aspects such as demographics (sex, age, family size, marital status, residential history, occupation, and education), self-reported medical conditions (diabetes mellitus, cardiovascular issues, and cancer), medications, and dietary supplements usage. To evaluate the physical activity levels of the participants, the Physical Activity Scale for the Elderly (PASE) questionnaire was employed.^37^ This questionnaire assessed the frequency and duration of physical activities performed in the past week across three domains: leisure-time activities (e.g., walking outdoors), household tasks (e.g., home maintenance), and work-related activities. Each activity was assigned a specific weight based on its frequency, and the total PASE score was calculated by summing up all activities. Higher PASE scores indicated greater levels of physical activity.^37^

###  Statistical analysis

 We extracted dietary patterns using exploratory factor analysis, which involved a principal component extraction method applied to 29 different food groups. To enhance interpretability, we utilized orthogonal varimax rotation to identify meaningful factors, which were selected for further investigation based on their eigenvalues (> 1.5) and the Scree test. These factors were labeled according to our interpretation of the data. Factor scores for each pattern were calculated by summing the intakes of food groups weighted by their factor loadings. Each participant was assigned a factor score for each identified dietary pattern. We divided participants into tertiles based on their dietary pattern scores and applied one-way analysis of variance (ANOVA) to assess differences in continuous variables across the tertiles. The chi-square test was used to evaluate the distribution of categorical variables across the tertiles. To assess the association of dietary patterns with obesity, hypertension, and cognitive function, we applied multivariable logistic regression analysis. Confounders were identified based on the literature and biological plausibility. The initial model was adjusted for basic confounders (age (continuous) and energy intake (continuous)). Subsequently, additional adjustments were made for demographic confounders (gender (male/female), marital status (single/married), supplement usage (yes/no), physical activity level (continuous), and education level (below high school diploma/high school diploma or above)) in the second model. In the final model, BMI was also included as a covariate to find an obesity-independent relationship between dietary patterns and investigated outcomes. The lowest tertile of dietary patterns was considered as the reference group for all analyses. Due to slight differences in the MMSE questionnaire used for literate and illiterate subjects, we performed a sensitivity analysis to test the robustness of our findings. We restricted our analyses to illiterate adults by excluding those who were literate. Statistical analyses were performed using IBM SPSS Statistics software version 19.0 (IBM Corp., Armonk, NY, USA). Two-sided *P* < 0.05 was considered statistically significant.

## Results

 The mean age of the study participants was 69.2 years (SD 7.29), of whom 37.7 percent were male. We identified 3 major dietary patterns including mixed, healthy, and unhealthy with factor analysis procedure (Table 1). These patterns accounted for 26% of the overall variance in dietary intakes. The mixed pattern was characterized by high intakes of refined grains, vegetables, red and processed meats, dried fruits, butter, cream, sour cream, eggs, onions, dairy, hydrogenated fats, legumes, salt, and boiled and fried potatoes. The healthy pattern was characterized by high intakes of olives, fruits, citrus fruits, fish, poultry, natural fruit juices, vegetables, nuts, dried fruits, whole grains, and legumes. The unhealthy pattern was characterized by snacks, fried potatoes, soft drinks, organ meats, salt, natural fruit juices, and mayonnaise.

**Table 1:** Factor-loading matrix for major dietary patterns^1^

**Food groups**	**Dietary patterns**		
	**Mixed**	**Healthy**	**Unhealthy**
Whole grain	-	0.25	-
Refined grain	0.61	-0.29	-
Boiled potatoes	0.34	-	-
Fried potatoes	0.38	-	0.59
Fruits	-	0.62	
Citrus fruit	-	0.59	-0.22
Natural fruit juices	0.21	0.42	0.30
Dried fruit	0.53	0.29	-
Vegetables	0.60	0.32	-
Legumes	0.36	0.21	-
Nuts	-	0.29	-
Onion	0.48	-	-
Vegetable oils	0.23	-	-
Olives	-	0.70	-
Red and processed meats	0.58	-	-
Organ meats	-	-	0.47
Fish	-	0.49	-
Poultry	-	0.43	-
Eggs	0.48	-	-
Total dairy	0.40	-	-
Hydrogenated fats	0.39	-0.21	-
Snacks	-	-	0.71
Sweets and desserts	-	-	-
Sugars	0.21	-	-
Soft drinks	-	-	0.48
Pizza	-	-	-
Salt	0.38	-	0.41
Mayonnaise	-	-	0.29
Butter, cream, and sour cream	0.52	-	0.21

^1^Values <0.20 were excluded for simplicity.

 General characteristics of the study participants across tertiles of dietary patterns are provided in Table 2. Individuals in the highest tertile of the mixed dietary pattern had a lower BMI and were more likely to be male and married compared to those in the lowest tertile. In comparison with the participants in the lowest tertile, those in the highest tertile of the healthy pattern were more likely to be married, educated, and use dietary supplements. Conversely, those in the upper tertile of the unhealthy dietary pattern were less likely to take dietary supplements than were those in the lowest. No significant difference was found in the distribution of other characteristics across categories of dietary patterns.

**Table 2:** Characteristics of study participants across tertile (T) categories of dietary pattern scores

	**Mixed pattern score**			P^*^	**Healthy pattern score**			P^*^	**Unhealthy pattern score**			P^*^
	**T1** **(n=112)**	**T2** **(n=113)**	**T3** **(n=112)**		**T1** **(n=112)**	**T2** **(n=113)**	**T3** **(n=112)**		**T1** **(n=112)**	**T2** **(n=113)**	**T3** **(n=112)**	
Age (years)	70.0 (7.83)	68.7 (6.92)	68.9 (7.08)	0.369	69.8 (7.24)	68.9 (7.08)	68.9 (7.56)	0.553	70.0 (8.23)	69.5 (6.64)	68.2 (6.84)	0.159
Male, n (%)	26 (23.2)	46 (40.7)	55 (49.1)	< 0.001	36 (32.1)	47 (41.6)	44 (39.3)	0.313	48 (42.9)	37 (32.7)	42 (37.5)	0.293
Married, n (%)	72 (64.3)	79 (69.9)	94 (83.9)	0.003	72 (64.3)	88 (77.9)	85 (75.9)	0.047	88 (78.6)	76 (67.3)	81 (72.3)	0.162
BMI (Kg/m^2^)	28.9 (5.14)	28.2 (4.47)	27.3 (4.28)	0.037	27.3 (4.02)	28.5 (4.58)	28.6 (5.27)	0.073	27.9 (4.61)	27.7 (4.25)	28.8 (5.09)	0.153
Educated, n (%)	10 (8.9)	15 (13.3)	16 (14.3)	0.428	3 (2.7)	9 (8.0)	29 (25.9)	< 0.001	11 (9.8)	13 (11.5)	17 (15.2)	0.455
History of diabetes, n (%)	33 (29.5)	27 (23.9)	22 (19.6)	0.229	21 (18.8)	30 (26.5)	31 (27.7)	0.237	35 (31.3)	25 (22.1)	22 (19.6)	0.103
History of hyperlipidemia, n (%)	42 (37.5)	30 (26.5)	31 (30.1)	0.147	26 (23.2)	41 (36.3)	36 (32.1)	0.094	39 (34.8)	32 (28.3)	32 (28.6)	0.488
Supplement use, n (%)	71 (63.4)	80 (70.8)	63 (56.3)	0.077	58 (51.8)	68 (60.2)	88 (78.6)	< 0.001	86 (76.8)	75 (66.4)	53 (47.3)	< 0.001

Abbreviations: BMI: body mass index. Data are presented as mean (standard deviation) or n (percent). ^*^Obtained by one-way ANOVA or chi-square, where appropriate.

 The dietary intakes of the participants across tertiles of dietary patterns are shown in Table 3. The energy and nutrient intakes of the participants in the highest tertile of the mixed pattern were significantly higher than those in the lowest tertile. Individuals in the highest tertile of the healthy pattern had significantly higher intakes of fiber, vitamin C, magnesium, calcium, and zinc. Greater adherence to unhealthy pattern was associated with higher intakes of total energy, protein, fat, carbohydrate, saturated fat, and zinc.

**Table 3:** Dietary intakes of study participants across tertile (T) categories of dietary pattern scores

	**Mixed pattern score**			P^*^	**Healthy pattern score**			P^*^	**Unhealthy pattern score**			P^*^
	**T1** **(n=112)**	**T2** **(n=113)**	**T3** **(n=112)**		**T1** **(n=112)**	**T2** **(n=113)**	**T3** **(n=112)**		**T1** **(n=112)**	**T2** **(n=113)**	**T3** **(n=112)**	
Total energy (kcal/d)	1499 (510)	1766 (555)	2393 (903)	< 0.001	1915 (846)	1814 (696)	1978 (744)	0.268	1848 (799)	1760 (679)	2099 (779)	0.002
Protein (g/d)	52.0 (21.9)	59.5 (26.7)	78.4 (32.7)	< 0.001	59.3 (24.7)	63.4 (35.7)	67.2 (26.7)	0.136	60.9 (30.3)	57.9 (22.1)	71.2 (33.7)	0.002
Fat (g/d)	37.5 (16.2)	47.9 (19.7)	63.2 (28.4)	< 0.001	48.8 (27.5)	46.6 (22.3)	53.2 (22.7)	0.114	46.7 (22.4)	45.6 (22.3)	56.3 (26.8)	0.001
Carbohydrate (g/d)	245 (98.0)	270 (97.2)	375 (163)	< 0.001	305 (154)	280 (113)	306 (134)	0.254	292 (138)	275 (120)	324 (142)	0.024
Dietary fiber (g/d)	10.6 (3.48)	12.5 (4.46)	17.3 (7.92)	< 0.001	11.9 (5.53)	12.9 (6.49)	15.6 (6.25)	< 0.001	13.7 (5.45)	13.0 (7.16)	13.7 (6.12)	0.609
Vitamin C (mg/d)	58.9 (30.8)	73.2 (36.9)	94.8 (62.2)	< 0.001	58.2 (35.6)	68.7 (32.3)	100 (59.8)	< 0.001	74.3 (33.4)	69.2 (39.2)	83.4 (63.8)	0.077
Magnesium (mg/d)	148 (70.2)	181 (74.9)	223 (95.0)	< 0.001	161 (81.6)	178 (77.1)	212 (91.9)	< 0.001	175 (73.2)	181 (95.2)	196 (87.9)	0.189
Saturated fat (g/d)	12.6 (6.33)	16.8 (7.19)	21.4 (9.73)	< 0.001	16.5 (9.25)	16.4 (8.91)	17.9 (7.68)	0.347	16.7 (8.72)	15.6 (7.86)	18.5 (9.10)	0.033
Calcium (mg/d)	675 (220)	845 (285)	1034 (385)	< 0.001	800 (367)	834 (329)	920 (303)	0.022	859 (358)	827 (334)	868 (319)	0.638
Zinc (mg/d)	5.05 (3.06)	6.28 (4.81)	7.78 (4.15)	< 0.001	5.30 (2.57)	6.67 (5.84)	7.14 (3.30)	0.003	6.10 (3.46)	5.82 (2.85)	7.20 (5.70)	0.035

Data are presented as mean (standard deviation). ^*^Obtained by one-way ANOVA.

Table 4 presents the odds ratios (ORs) for obesity, hypertension, and cognitive function across tertile categories of dietary patterns. There was an inverse association between the mixed dietary pattern and abdominal obesity either before (OR: 0.48, 95% CI: 0.28-0.82) or after (OR: 0.39, 95% CI: 0.20-0.76) controlling for potential confounders. A non-significant association was found between adherence to the mixed dietary pattern and general obesity in the crude model (OR: 0.58, 95% CI: 0.33-1.03). This association became significant after controlling for the potential confounders (OR: 0.49, 95% CI: 0.24-0.99). There was a significant positive association between unhealthy dietary pattern and abdominal obesity either before (OR: 1.99, 95% CI: 1.15-3.44) or after (OR: 1.90, 95% CI: 1.05-3.44) adjusting for confounders. A higher score of mixed pattern was associated with lower odds of hypertension either before (OR: 0.35, 95% CI: 0.19-0.64) or after (OR: 0.39, 95% CI: 0.20-0.78) adjusting for confounders. Moreover, a significant positive association was observed between unhealthy dietary pattern and hypertension (OR: 1.78, 95% CI: 1.01-3.15). This association remained significant after taking potential confounders into account (OR: 1.86, 95% CI: 1.01-3.43). There was no significant association between dietary patterns and cognitive function in the crude and fully-adjusted models. In the sensitivity analyses, the associations between dietary patterns and cognitive function remained unchanged after excluding illiterate participants (data not shown).

**Table 4:** Multivariable-adjusted odds ratios for obesity, hypertension, and cognitive function across tertile (T) categories of dietary pattern scores

	**Mixed pattern score**			***P*** ** for trend**	**Healthy pattern score**			***P*** ** for trend**	**Unhealthy pattern score**			***P*** ** for trend**
	**T1** **(n=112)**	**T2** **(n=113)**	**T3** **(n=112)**		**T1** **(n=112)**	**T2** **(n=113)**	**T3** **(n=112)**		**T1** **(n=112)**	**T2** **(n=113)**	**T3** **(n=112)**	
General obesity												
Crude	1	0.72 (0.41-1.25)	0.58 (0.33-1.03)	0.062	1	1.38 (0.78-2.45)	1.35 (0.76-2.39)	0.312	1	0.95 (0.53-1.68)	1.33 (0.76-2.33)	0.313
Model 1	1	0.59 (0.32-1.06)	0.40 (0.20-0.80)	0.008	1	1.30 (0.72-2.37)	1.24 (0.68-2.26)	0.490	1	0.94 (0.52-1.73)	1.17 (0.65-2.12)	0.599
Model 2	1	0.68 (0.37-1.26)	0.49 (0.24-0.99)	0.044	1	1.53 (0.82-2.87)	1.41 (0.72-2.76)	0.312	1	0.87 (0.47-1.64)	1.14 (0.61-2.16)	0.681
Abdominal obesity												
Crude	1	0.80 (0.47-1.39)	0.48 (0.28-0.82)	0.007	1	1.41 (0.83-2.39)	1.44 (0.85-2.45)	0.176	1	0.95 (0.56-1.60)	1.99 (1.15-3.44)	0.015
Model 1	1	0.71 (0.40-1.24)	0.37 (0.20-0.69)	0.002	1	1.35 (0.79-2.31)	1.39 (0.81-2.40)	0.226	1	0.91 (0.53-1.55)	1.91 (1.09-3.35)	0.028
Model 2	1	0.76 (0.42-1.36)	0.39 (0.20-0.76)	0.006	1	1.33 (0.76-2.33)	1.38 (0.76-2.50)	0.274	1	0.89 (0.52-1.56)	1.90 (1.05-3.44)	0.039
Hypertension												
Crude	1	0.48 (0.26-0.89)	0.35 (0.19-0.64)	0.001	1	0.97 (0.54-1.73)	0.61 (0.35-1.08)	0.085	1	1.45 (0.83-2.52)	1.78 (1.01-3.15)	0.045
Model 1	1	0.49 (0.27-0.91)	0.35 (0.18-0.67)	0.002	1	0.98 (0.54-1.76)	0.63 (0.36-1.12)	0.109	1	1.44 (0.82-2.52)	2.02 (1.12-3.63)	0.018
Model 2	1	0.53 (0.28-0.99)	0.37 (0.19-0.74)	0.005	1	1.09 (0.60-1.98)	0.68 (0.37-1.26)	0.228	1	1.35 (0.76-2.39)	1.89 (1.03-3.47)	0.040
Model 3	1	0.53 (0.28-1.01)	0.39 (0.20-0.78)	0.007	1	1.04 (0.57-1.90)	0.64 (0.35-1.20)	0.169	1	1.38 (0.78-2.45)	1.86 (1.01-3.43)	0.045
Cognitive function												
Crude	1	0.27 (0.05-1.33)	0.27 (0.05-1.34)	0.072	1	0.99 (0.24-4.06)	0.74 (0.16-3.40)	0.707	1	0.32 (0.06-1.61)	0.49 (0.12-1.99)	0.266
Model 1	1	0.27 (0.05-1.42)	0.20 (0.03-1.40)	0.065	1	1.11 (0.26-4.79)	0.74 (0.15-3.55)	0.718	1	0.39 (0.07-2.05)	0.66 (0.15-2.90)	0.514
Model 2	1	0.32 (0.06-1.78)	0.34 (0.05-2.47)	0.182	1	1.25 (0.27-5.69)	0.75 (0.14-4.10)	0.766	1	0.30 (0.06-1.70)	0.56 (0.12-2.69)	0.412
Model 3	1	0.35 (0.06-1.92)	0.32 (0.04-2.31)	0.175	1	1.42 (0.30-6.64)	0.90 (0.17-4.95)	0.937	1	0.26 (0.05-1.48)	0.45 (0.09-2.26)	0.289

Abbreviations: BMI: body mass index; OR: odds ratio; CI: confidence interval. Data are presented as OR and 95% CI. Model 1: Adjusted for age and energy. Model 2: Additional adjustment for gender, marital status, supplement use, physical activity, and education level. Model 3: Additional adjustment for BMI. ^*^Obtained from logistic regression.

## Discussion

 In the current study, we found that greater adherence to mixed dietary patterns was inversely linked to hypertension, general obesity, and abdominal obesity. Moreover, unhealthy pattern was associated with higher odds of abdominal obesity and hypertension. However, there was no significant association between dietary patterns and cognitive function.

 Obesity, hypertension, and cognitive disorders are the major public health challenges around the world. Diet and lifestyle undisputedly play a major part in the development and progression of such conditions. Over the past years, multiple studies have investigated the relation of dietary components with obesity, hypertension, and cognitive function. However, available evidence linking the whole diet to disease risk, especially in older adults is scarce. Unlike Western countries, people in the Middle East consume meals that contain large amounts of refined grains and most of the energy source comes from carbohydrates. Therefore, the study of dietary patterns in this area can provide more evidence regarding diet-disease relations. To the best of our knowledge, this study is the first to examine the association of major dietary patterns with obesity, hypertension, and cognitive function in the elderly population in the Middle East region.

 We found that greater adherence to unhealthy and mixed dietary patterns was associated with higher and lower odds of abdominal obesity. Moreover, there was a significant inverse association between mixed dietary pattern and general obesity. Results from the Baltimore Longitudinal Study of Aging suggested that a diet high in reduced-fat dairy products and fiber-rich foods was associated with smaller increases in WC for both men and women, as well as smaller increases in BMI for women.^38^ In another study, a vegetable-fruit dietary pattern was associated with a reduced likelihood of being overweight or obese, particularly among women. Conversely, a meat-processed dietary pattern was linked to higher odds of overweight and obesity in both genders.^39^ In an observational investigation among Brazilian older adults, adherence to the prudent (including fruits, vegetables, and meat) and Mediterranean (comprising fruits, vegetables, olive oil, and nuts) dietary patterns was protectively linked to general and abdominal obesity. However, sweets and fats were not significantly associated with typical Brazilian and traditional dietary patterns.^40^ The lack of significant association of healthy and unhealthy dietary patterns with general obesity could be related to the nature of BMI. An important methodological limitation in obesity research that has been underexplored is that BMI is an imperfect measure of obesity.^41,42^ Although BMI measures overweight relative to height, it does not differentiate between fat mass and lean body mass; therefore, when using BMI as a measure, inaccurate assessment of adiposity could occur.^43^ In this study, unlike the mixed food pattern, the healthy pattern was not related to abdominal obesity. This finding was unexpected and needs to be investigated in future studies to determine which components of the mixed dietary pattern are inversely related to abdominal obesity.

 In the present study, higher adherence to unhealthy and mixed dietary patterns was associated with higher and lower odds of hypertension. According to China Health and Nutrition Survey, following a modern dietary pattern with a high consumption of fruits and dairy products was linked to lower SBP. Conversely, the meat-centric dietary pattern was associated with higher DBP and an increased risk of hypertension.^44^ In a further study, the “fruit and milk” pattern was associated with a lower prevalence of both pre-hypertension and hypertension among middle-aged and elderly Chinese men in Shanghai.^45^ In a cross-sectional study, it was found that individuals with a drinking pattern score, characterized by moderate to high alcohol intake and salted fermented seafood consumption, had a significantly higher prevalence of prehypertension or hypertension. Additionally, men following a Western dietary pattern had a higher prevalence of hypertension. Interestingly, the whole food pattern did not show any significant association with either prehypertension or hypertension.^46^ Overall, the findings of this study and previous studies highlight the importance of an unhealthy diet in increasing the risk of hypertension. Several components of the dietary patterns could explain the associations obtained for obesity and hypertension. In three large prospective cohorts, higher intake of French fries (loaded strongly in unhealthy pattern) was associated with an increased risk of developing hypertension.^47^ Moreover, a significant positive association was seen between higher consumption of sugar and artificially sweetened beverages and risk of hypertension in a dose-response meta-analysis of prospective observational studies.^48^ Sugar-sweetened beverages are associated with increased blood glucose levels, increased appetite, and weight gain.^49^ These beverages are the main source of fructose in the diet, and high consumption of fructose increases the synthesis of triglycerides in the liver and causes dyslipidemia, abdominal obesity, and insulin resistance.^49^ Data from epidemiological studies have provided an inverse association between dairy intake (loaded strongly in mixed dietary pattern) and hypertension.^50^ Moreover, available evidence suggests that dairy products, especially fermented ones, are associated with a reduced risk of obesity, which is one of the main causes of hypertension.^51^ Some, but not all, studies also pointed toward fruits and vegetables intake, as a protective factor for hypertension.^52^ Moreover, a significant reduction in SBP and DBP was reported following soy consumption in a meta-analysis of randomized clinical trials.^53^ Similarly, total legume consumption among the over-65s was associated with a reduced risk of hypertension.^54^ Fruits, vegetables, dairy products, and legumes contain fiber, vitamins and minerals, proteins, antioxidants, phenolic compounds, and unsaturated fatty acids. Minerals such as potassium, calcium, and magnesium can reduce peripheral vascular resistance —and, consequently, blood pressure— by facilitating the synthesis of prostacyclin and nitric oxide, as well as by reducing the level of angiotensin II. Moreover, vegetables are a rich source of folic acid, which lowers plasma homocysteine levels. Evidence from animal and human studies has shown that greater serum levels of homocysteine are associated with an increased risk of hypertension. Moreover, several studies have documented that higher consumption of plant proteins is associated with favorable changes in blood pressure.^55,56^

 In the current study, major dietary patterns were not associated with cognitive function of the participants. In contrast to our findings, a cross-sectional study examined the association of dietary patterns with cognitive impairment among Chinese elderly. The investigators identified four dietary patterns and found that participants who scored highly on food pattern 1 (characterized by high consumption of legumes, vegetables, fruits, milk and dairy products, and nuts, and low consumption of noodles and cereals) exhibited better direction, memory, and language function.^57^ Findings from another study suggested that a “plant foods and fish” pattern (characterized by vegetables, soy products, fruit, and fish) may lead to favorable changes in cognitive function of older Japanese people. However, neither the rice and miso soup nor the animal food pattern was associated with cognitive function.^58^ In a longitudinal study conducted by Xu et al^59^ among Chinese elderly, three dietary patterns including traditional (rice, pork, and fish), protein-rich (high consumption of milk, eggs, and soy milk), and starch-rich (high consumption of salty vegetables and legumes) were identified. Protein-rich food pattern was significantly related to greater cognitive scores and verbal memory scores, while starch-rich food pattern was associated with lower overall and verbal cognitive memory scores. Compared to previous studies, a relatively small number of cases with cognitive impairment were included in our analysis, which reduces the power of the study to find significant associations. Therefore, large additional studies are needed to fully understand the association between dietary patterns and cognitive function in the Middle East region. Moreover, the disagreements between our findings and other studies might be partially explained by differences in methodologies, populations, identified dietary patterns, and analytic approaches across studies. For example, logistic regression analysis was applied to investigate the association of dietary patterns with cognitive function in our study. However, Okubo et al^58^ and Su et al^57^ used multiple linear regression analysis.

 The present studyhas several strengths. First, the use of dietary patterns allowed us to investigate the interaction among synergistic dietary components. Second, we controlled for main confounders in our statistical analyses to obtain an independent association between the dietary patterns and odds of study outcomes. Third, valid questionnaires were used to evaluate food intake and other variables. However, some limitations should be considered when interpreting our findings. First, we cannot infer a causal association of dietary patterns with obesity, hypertension, and cognitive function due to the observational nature of our study. Therefore, further prospective studies are needed to establish causality. Second, the semi-quantitative FFQ is an imperfect tool for assessing dietary intake; therefore, measurement errors and random misclassification of dietary intakes may have occurred. Third, we cannot rule out unmeasured and residual confounding due to the cross-sectional nature of the study. Fourth, the sample size was not large enough to examine the relationship stratified by gender and other demographic characteristics. Fifth, the exploratory dietary patterns obtained from the factor analysis are specific to the studied population, and as a result, the contribution of the findings of a single study to evidence-based recommendations is limited. Factor analysis was applied to measure dietary patterns which has several limitations. For example, in such cases subjective decisions are made regarding the number of patterns, it is unclear which food items characterize the pattern, and only a low to moderate proportion of intake are explained. Finally, our findings are not generalizable to young adults or other populations.

 Our findings support recommendations on increasing consumption of certain types of healthy foods (loaded strongly in mixed dietary pattern) for improving health. Future studies could explore targeted dietary interventions within Middle Eastern populations, considering the cultural dietary practices that may influence health outcomes in older adults. Additionally, public health programs could focus on promoting adherence to dietary patterns associated with improved health outcomes, particularly in culturally relevant ways.

## Conclusion

 The current study provides evidence for an inverse association of mixed pattern with general obesity, abdominal obesity, and hypertension. Moreover, unhealthy pattern is associated with higher odds of obesity and hypertension Several unexpected results in this study, including the non-significant association of healthy dietary pattern with obesity and hypertension, merit more investigation.

## Competing Interests

 The authors declare no conflict of interest.

## Ethical Approval

 This study was conducted according to the guidelines laid down in the Declaration of Helsinki and all procedures involving human subjects/patients were approved by the Bioethics Committee of Tabriz University of Medical Sciences, Tabriz, Iran (IR.TBZMED.REC.1401.575).
